# Author Correction: Physical networks as network-of-networks

**DOI:** 10.1038/s41467-024-52590-1

**Published:** 2024-09-19

**Authors:** Gábor Pete, Ádám Timár, Sigurdur Örn Stefánsson, Ivan Bonamassa, Márton Pósfai

**Affiliations:** 1 HUN-REN Alfréd Rényi Institute of Mathematics, Budapest, Hungary; 2 Department of Stochastics, Institute of Mathematics, Budapest University of Technology and Economics, Műegyetem rkp. 3., H-1111 Budapest, Hungary; 3 University of Iceland, Reykjavík, Iceland; 4 Department of Network and Data Science, Central European University, Vienna, Austria

**Keywords:** Complex networks, Applied mathematics

Correction to: *Nature Communications* 10.1038/s41467-024-49227-8, published online 7 June 2024

In this article, the affiliation details for Gábor Pete and Ádám Timár were incorrectly given as ‘Alfréd Rényi Institute of Mathematics, Budapest, Hungary’ but should have been ‘HUN-REN Alfréd Rényi Institute of Mathematics, Budapest, Hungary’. In addition, an affiliation detail for Gábor Pete was incorrectly given as ‘Budapest University of Technology and Economics, Budapest, Hungary’ but should have been ‘Department of Stochastics, Institute of Mathematics, Budapest University of Technology and Economics, Műegyetem rkp. 3., H-1111 Budapest, Hungary’. The original article has been corrected.

