# Supplementary material for: Physical networks as network-of-networks
Source: Nat Commun. 2024 Jun 7;15:4882. doi: 10.1038/s41467-024-49227-8 (PMC11161514; doi:10.1038/s41467-024-49227-8)
Supplement: Supplementary file 1 — Supplementary Information [file 41467_2024_49227_MOESM1_ESM.pdf]

Supplementary Information – Physical networks as network-of-networks

Gábor Pete,[1,2,*] Ádám Timár,[1,3] Sigurdur Örn Stefánsson,[3]
Ivan Bonamassa,[4] and Márton Pósfai[4,†]

[1] *Alfréd Rényi Institute of Mathematics, Budapest, Hungary*
[2] *Budapest University of Technology and Economics, Budapest, Hungary*
[3] *University of Iceland, Reykjavík, Iceland*
[4] *Department of Network and Data Science, Central European University, Vienna, Austria*

CONTENTS

S1. Physical node growth algorithms 2

S2. Empirical physical networks 17

Supplementary references 21

* gabor.pete@renyi.hu
† posfaim@ceu.edu

S1. PHYSICAL NODE GROWTH ALGORITHMS

Relying on the network-of-networks representation of physical networks, we introduced a simple model of physical network evolution in the main text. In the model, physical nodes are added sequentially and grow following a random trajectory until they connect to the existing network. The analytical description of the model imposes mild restrictions on the random trajectory that grows the physical nodes: the trajectory must be described by a fractal dimension $d_\mathrm{f} \in [1, d]$ (where d is the embedding dimension) and the random trajectories need to have the property that if the boxes around two random walk pieces intersect, then with uniformly positive probability the pieces also intersect. This latter condition requires a level of isotropy, at least on average. For a simple counterexample, consider nodes embedded in a two dimensional square lattice always growing along the horizontal axis; in this case two nodes can run parallel to each other with overlapping bounding boxes without intersecting, and the resulting network will be a collection of disconnected chains.

The analytical description of the model presented in the main text predicts that independent of the details of the physical node trajectories, the degree distribution of the emergent physical network has a power law tail; however the exact value of the degree exponent γ does depend on d_f. Specifically, we make the following predictions:

- The total volume of the network grows as

$$V_t \sim L^d \left(\frac{t}{L^d}\right)^{1-d_\mathrm{f}/d}, \tag{S1}$$

  where L is the linear dimension of the embedding d dimensional square lattice.

- For large nodes, the degree of the physical nodes is proportional their volume

$$k_t(N) = 1 + \frac{v_t}{L^d}\sum_{s=t+1}^{N} v_s^{d/d_\mathrm{f}-1} \sim v_t \cdot \left(\frac{N}{L^d}\right)^{\frac{d}{d_\mathrm{f}}}. \tag{S2}$$

- The tail of the degree distribution is characterized by a power law $P(k) \sim k^{-(\gamma-1)}$ with exponent

$$\gamma = 1 + \frac{d}{d_\mathrm{f}}, \tag{S3}$$

  for $2d_\mathrm{f} > d$, for $2d_\mathrm{f} \leq d$ we are in the meanfield regime with $\gamma_\mathrm{MF} = 3$.

In the remainder of this section we compare the above predictions to numerical simulations using a wide range of random node trajectories, in addition to loop-erased random walk node trajectories studied in the main text.

S1.1. Meanfield node growth: random point cloud

In the meanfield regime ($2d_{\rm f} < d$) the fractal dimension $d_{\rm f}$ of the physical nodes is so small that even if the boxes containing two nodes $\mathcal{V}_i$ and $\mathcal{V}_j$ overlap, nodes i and j avoid each other with high probability. In this regime the network evolution follows the characteristics of the network evolution in infinite dimensions $d = \infty$, i.e., the substrate $\mathcal{S}$ is a complete graph. To simulate meanfield dynamics, we can equivalently grow physical nodes by random jumps:

- We embed the growing network in a finite d-dimensional lattice.
- Each physical node $\mathcal{V}_t$ is grown by adding a uniformly random lattice site to $\mathcal{V}_t$.

Note that the lattice sites that we sequentially add to $\mathcal{V}_t$ are not necessarily adjacent, and $\mathcal{V}_t$ is typically not a connected sub-graph of $\mathcal{S}$.

Figure S1 shows the physical layout $\mathcal{P}$ and the combinatorial network $\mathcal{G}$ of an example network generated using random jump nodes. Figure S2 compares the analytical predictions to simulations, finding excellent agreement.

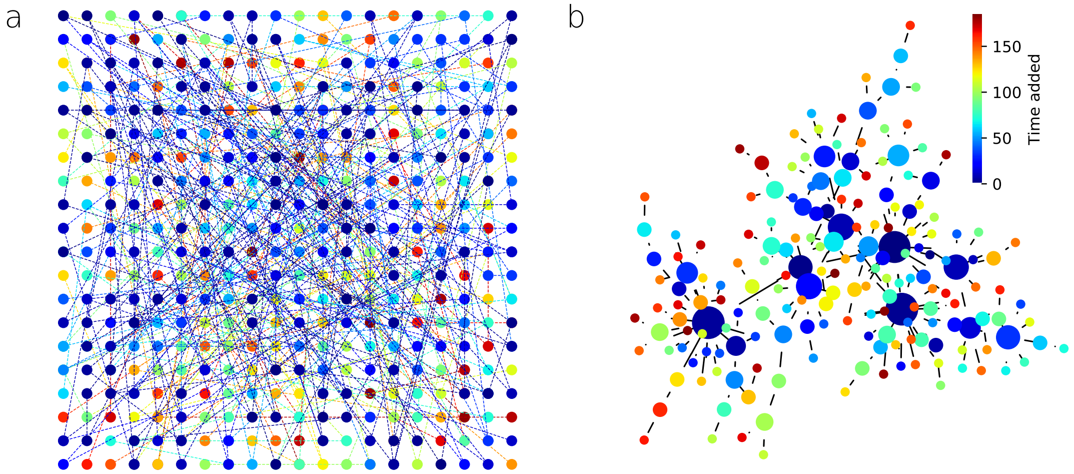

FIG. S1. **Example network generated with random jump nodes.** (a) The physical layout $\mathcal{P}$ of a saturated network embedded in a 20×20 square lattice with periodic boundary conditions. The color of physical nodes indicate the time they were added. Sequential lattice sites in a physical node trajectory $\mathcal{V}_t$ (connected by dashed lines) are typically non-adjacent. (b) The corresponding combinatorial network $\mathcal{G}$. Node sizes are a linear function of the logarithm of their degrees.

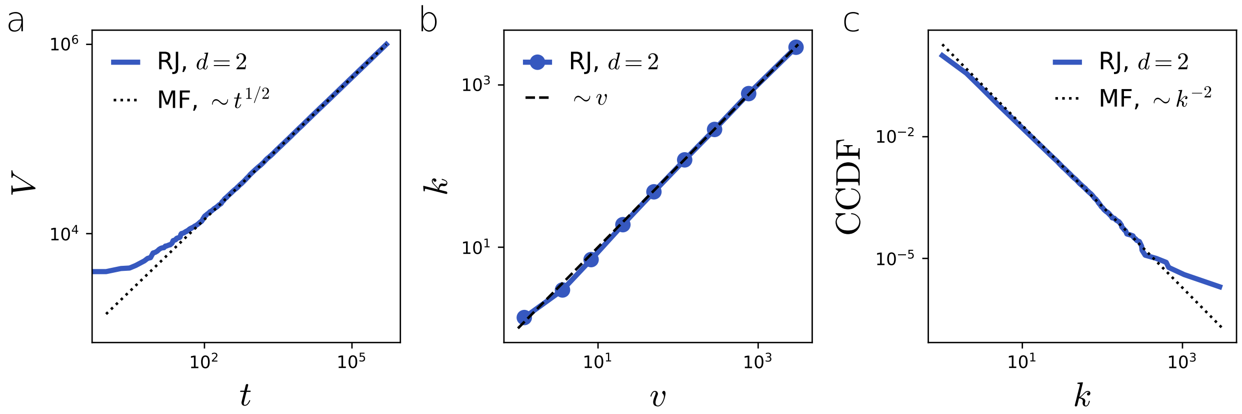

FIG. S2. **Random jump networks.** (a) Evolution of the total volume of the network V_t. (b) Correlations between node volume v and node degree degree k. (c) Complementary cumulative distribution of the node degree. (a-c) We compare the predictions of Eqs. (S1),(S2) and (S3) to numerical simulations, finding near perfect agreement. The lines indicate results for a single saturated network embedded on d-dimensional square lattice with periodic boundaries and side length L, where L is chosen such that $L^d \approx 10^6$.

S1.2. Simple random walk

We also generate physical nodes using trajectories of simple random walks (SRW). In this case, a physical node $\mathcal{V}_i$ is allowed to intersect itself and halts its growth once it hits another $\mathcal{V}_j$ $(i \neq j)$. In other words, volume exclusion is only imposed between the trajectories distinct nodes, i.e., $\mathcal{V}_i \cap \mathcal{V}_j$ still holds for $i \neq j$. The fractal dimension of SRWs is $d_{\mathrm{f}} = 2$ for embedding dimensions $d \leq 2$, meaning that for $d \leq 4$ we are in the meanfield regime. Embedding dimension $d = 2$ also represents a special case $d = d_{\mathrm{f}}$, for which Eq. (S1) predicts that the exponent of the total volume growth is $1 - d_{\mathrm{f}}/d = 0$, and we expect that other effects not captured by the theory to become relevant. For example, since each new physical node starts from an unoccupied site, the minimum node volume in simulations is one, i.e., $|\mathcal{V}_t| \leq 1$, which becomes relevant if the predicted node volume falls below unity. Figure S3 shows the physical layout $\mathcal{P}$ and the combinatorial network $\mathcal{G}$ of an example network generated using SRWs. Figure S4 compares the analytical predictions to simulations, finding excellent agreement.

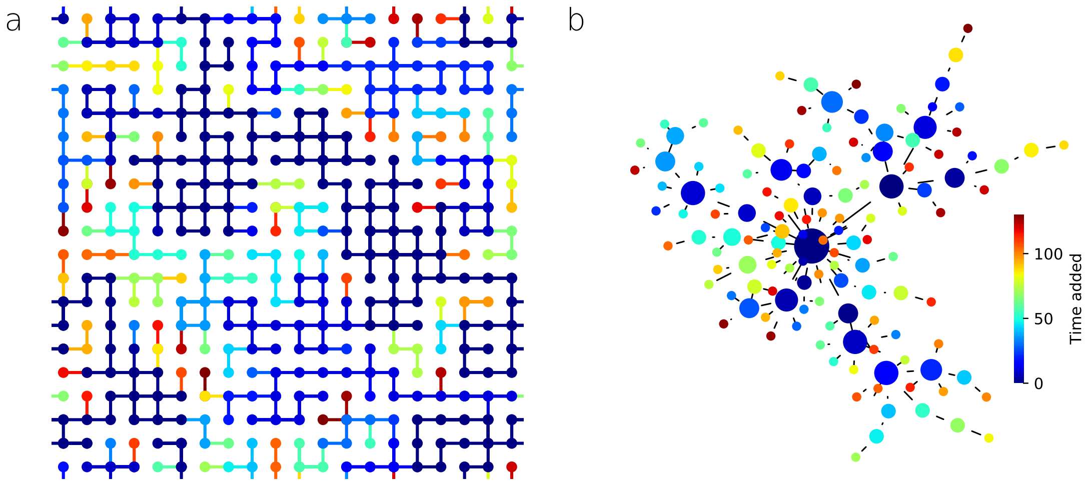

FIG. S3. **Example network generated with SRWs.** (a) The physical layout $\mathcal{P}$ of a saturated network embedded in a 20×20 square lattice with periodic boundary conditions. The color of physical nodes indicate the time they were added. A SRW trajectory may intersect itself. (b) The corresponding combinatorial network $\mathcal{G}$. Node sizes are a linear function of the logarithm of their degrees.

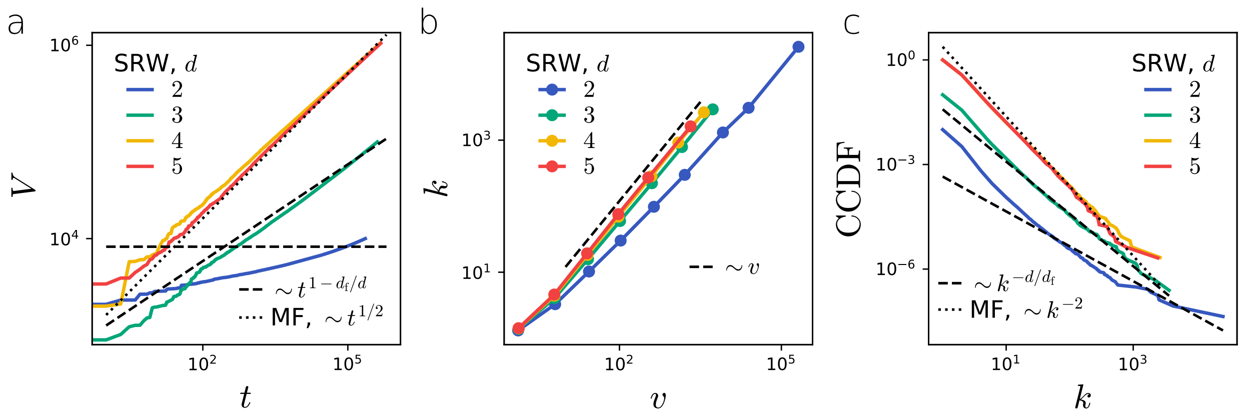

FIG. S4. **SRW networks.** (a) Evolution of the total volume of the network V_t. (b) Correlations between node volume v and node degree degree k. (c) Complementary cumulative distribution of the node degree. (a-c) We compare the predictions of Eqs. (S1),(S2) and (S3) to numerical simulations, finding excellent agreement. The lines indicate results for a single saturated network embedded on d-dimensional square lattice with periodic boundaries and side length L, where L is chosen such that $L^d \approx 10^6$.

S1.3. Kinetic self-avoiding random walk

We also test our predictions against networks grown from kinetic self-avoiding random walk (KSAW) trajectories. Self-avoiding random walks were introduced to model polymer chains, and are most often studied in an equilibrium setting, i.e., in an equilibrium ensemble all self-avoiding trajectories of a given length have the same weight [1]. Here, we grow physical nodes using the kinetic version, also called genuine self-avoiding walks: a walker starts from a random lattice site and always moves to a uniformly chosen adjacent site that it has not visited before [2]. Such random walks seem as a natural choice for our network model; however, the KSAW traps itself in two and three dimensions after a finite number of steps. In large lattices ($L \to \infty$), the expected maximum volume of KSAW trajectories is approximately $v_2^{\max} \approx 71$ in two dimensions and $v_3^{\max} \approx 4000$ in three dimensions S5 [3]. This means that it becomes impossible to grow physical networks using our original model for $L \ll v^{\max}$ embedding lattices, as a result we only simulate networks for $d \leq 3$ on lattices with $L^d \approx 10^6$ sites.

In three dimensions the fractal dimension of SAWs is $d_\mathrm{f} \approx 1.7$, above three dimensions the probability of self-intersection asymptotically becomes zero and SAWs are characterized by the fractal dimension of SRWs, i.e., $d_\mathrm{f} = 2$ for $d \leq 4$ [4]. This means that KSAW networks are in the meanfield regime for dimensions $d > 3$. Figure S6 shows the physical layout $\mathcal{P}$ and the combinatorial network $\mathcal{G}$ of an example network generated using KSAWs. Figure S7 compares the analytical predictions to simulations, finding excellent agreement.

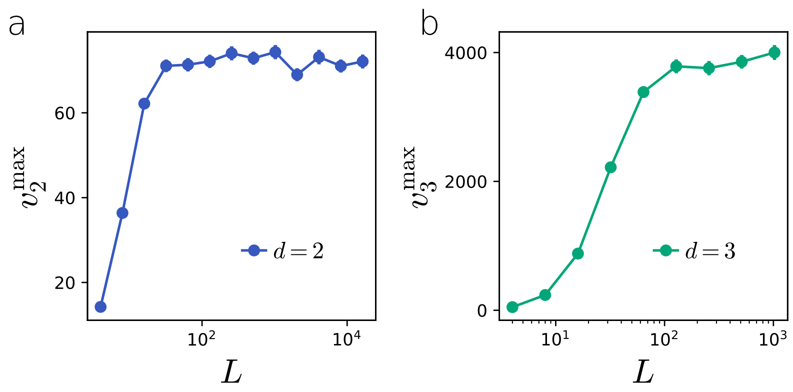

FIG. S5. **Self-trapping of KSAW trajectories.** (a) Expected maximum volume of a KSAW trajectory for embedding dimension $d = 2$ and (b) embedding dimension $d = 3$. (a-b) We grow KSAW trajectories in isolation until they get trapped, i.e., all sites adjacent to the walker have been visited before, and we measure their final volume $v^{\max}$ as a function of lattice size L. On finite lattices, all walkers eventually get trapped, the fact that $v^{\max}$ plateaus and becomes indpendent of L indicates that KSAWs trap themselves in the $L \to \infty$ limit for $d = 2$ and $d = 3$. Markers represent an average of 1000 independent runs and the errorbars provide the standard error of the mean.

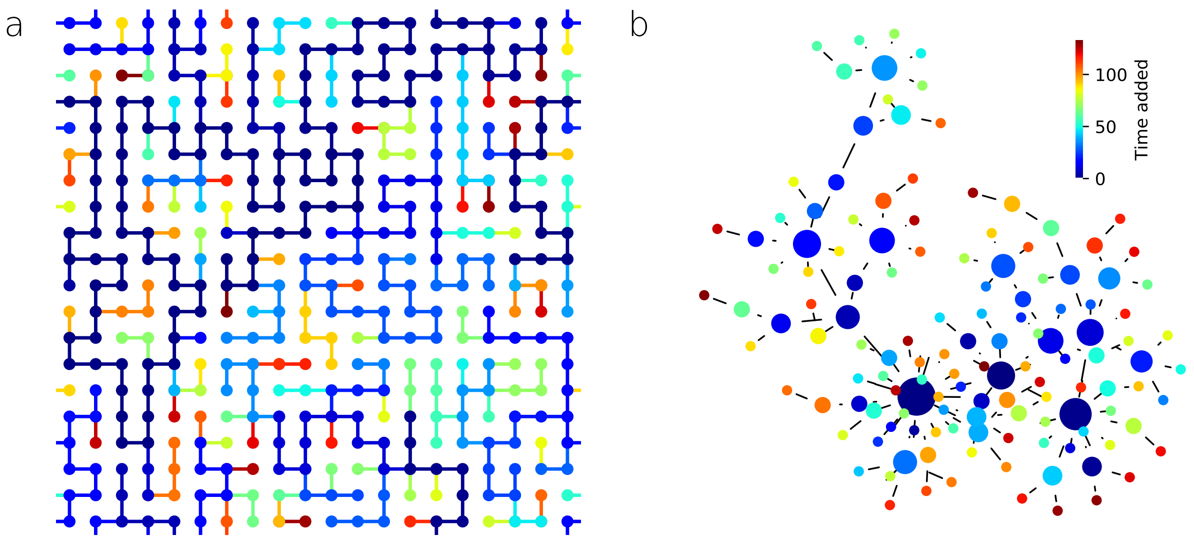

FIG. S6. **Example network generated with KSAWs.** (a) The physical layout $\mathcal{P}$ of a saturated network embedded in a 20×20 square lattice with periodic boundary conditions. The color of physical nodes indicate the time they were added. A KSAW trajectory traps itself after $v_2^{\max} \approx 71$ steps in 2 dimensions; therefore, we would not be able to generate KSAW networks for $L \ll 71$ for $d = 2$. In this simulation, we restart the growth of nodes that got trapped. (b) The corresponding combinatorial network $\mathcal{G}$. Node sizes are a linear function of the logarithm of their degrees.

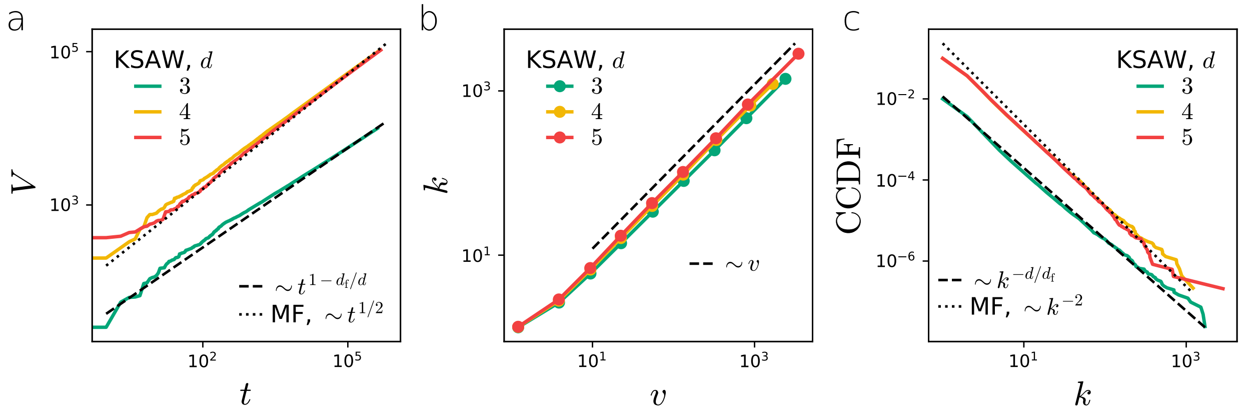

FIG. S7. **KSAW networks.** (a) Evolution of the total volume of the network V_t. (b) Correlations between node volume v and node degree degree k. (c) Complementary cumulative distribution of the node degree. (a-c) We compare the predictions of Eqs. (S1),(S2) and (S3) to numerical simulations, finding excellent agreement. The lines indicate results for a single saturated network embedded on d-dimensional square lattice with periodic boundaries and side length L, where L is chosen such that $L^d \approx 10^6$.

S1.4. Random ray

Finally, we generate random nodes tracing random rays (RR): we grow nodes from a random starting lattice site in a uniform random direction parallel to one of the axis, nodes grow until they hit an already existing node. The RR growth process produces physical nodes with $d_{\mathrm{f}} = 1$ for any embedding dimension $d \leq 2$; therefore the network evolution is always in the meanfield regime. We build the networks on lattices with periodic boundary conditions, hence at the early stages of network growth, the RR trajectories often miss existing nodes, and instead return the their starting points and collide with themselves, forming rings. In these cases, we kept the trajectories in $\mathcal{P}$ and added an isolated node to the combinatorial network $\mathcal{G}$; therefore the final network breaks into many components (Fig. S8).

Figure S9 shows the physical layout $\mathcal{P}$ and the combinatorial network $\mathcal{G}$ of an example network generated using RRs. Figure S8 compares the analytical predictions to simulations, finding excellent agreement, with two caveats: First, for $d = 2$, the numerically measured degree distribution is in excellent agreement with the meanfield predicition (Fig. S10)c; however, the evolution of the total volume V_t and the degree-volume correlations somewhat deviate from the prediction. We explain this by the observation that for $d = 2$ long nodes can be blocked from receiving connections by other nodes running parallel to them, for example, the horizontal light blue node in Fig. S9a remains isolated due to the two parallel nodes adjacent to it. For higher embedding dimensions $d > 2$, this is an increasingly unlikely event. Second, for $d > 2$, at early stages of the network the embedding lattice is sparsely populated and nodes are likely to miss each other, This means that Eq. (1) of the main text holds only after a sufficient density of nodes is reached. This leads to a deviation from the predicted power law growth of the total volume for small t (Fig. S10a) and introduces a cutoff in the degree distribution for high k (Fig. S10c).

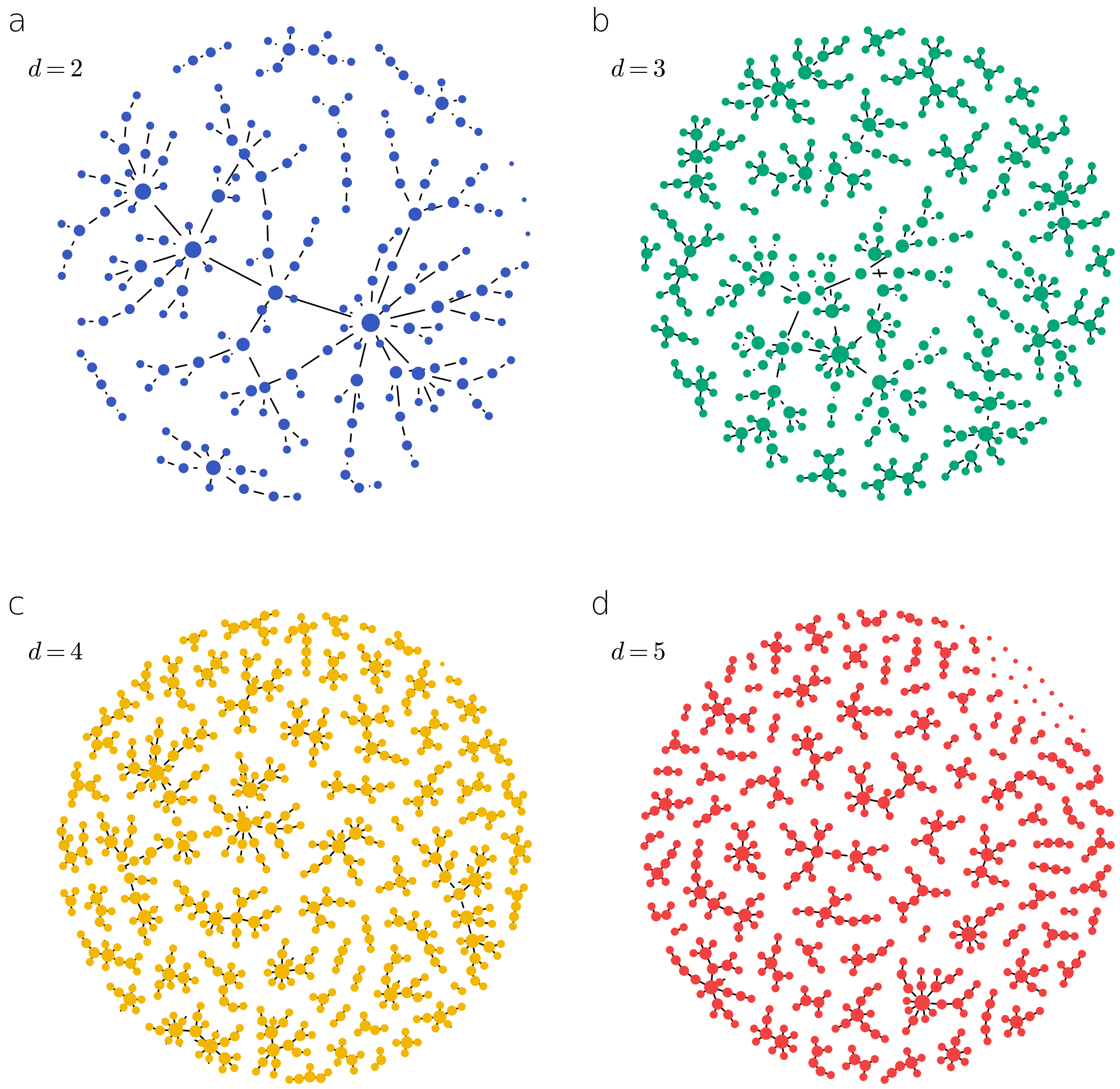

FIG. S8. **RR combinatorial networks.** (a) We show typical combinatorial networks for dimension $d = 2$, (b) $d = 3$, (c) $d = 4$ and (d) $d = 5$. (a-d) At early stages of the network evolution, node trajectories may miss other nodes and loop around to form a ring. Each of these nodes forms a connected component in the final combinatorial network $\mathcal{G}$. Each combinatorial network $\mathcal{G}$ corresponds to a saturated physical layout $\mathcal{P}$ embedded in a d-dimensional square lattice with periodic boundaries and side length L, where L is chosen such that $L^d \approx 10^3$.

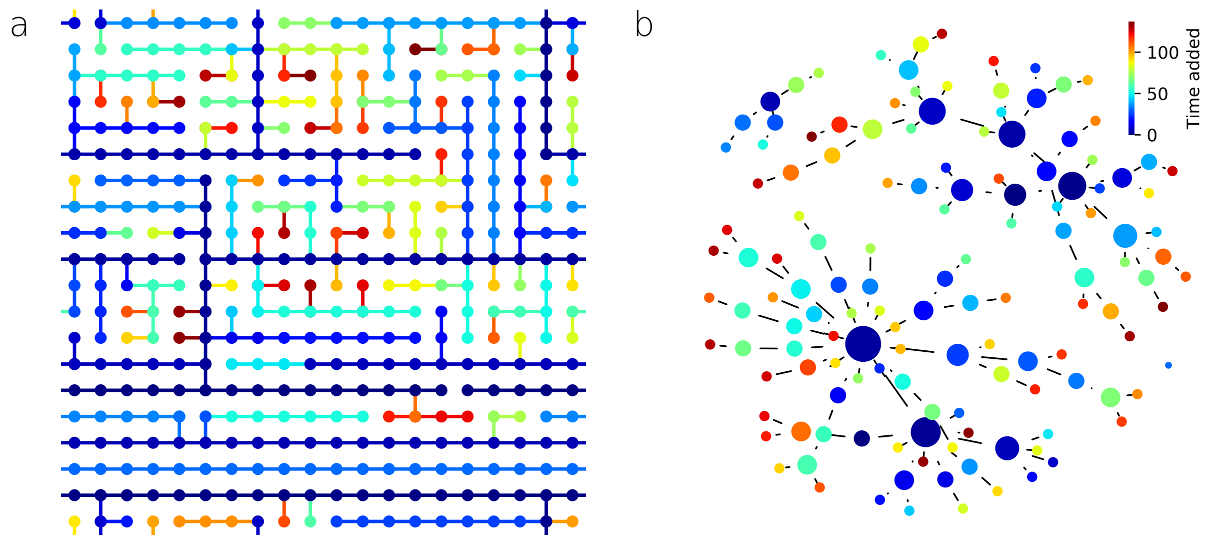

FIG. S9. **Example network generated with RRs.** (a) The physical layout $\mathcal{P}$ of a saturated network embedded in a 20×20 square lattice with periodic boundary conditions. The color of physical nodes indicate the time they were added (note that the color of nodes added at consecutive time steps is indistinguishable). Nodes added early may loop around and form a ring. For $d = 2$, nodes with high volume may be shielded from receiving connections by adjacent nodes running parallel to them (light blue horizontal node). (b) The corresponding combinatorial network $\mathcal{G}$. The network has three connected components: two grown from the two horizontal dark blue nodes that form rings, and an isolated node corresponding to the light blue node shielded by its neighbors. Node sizes are a linear function of the logarithm of their degrees.

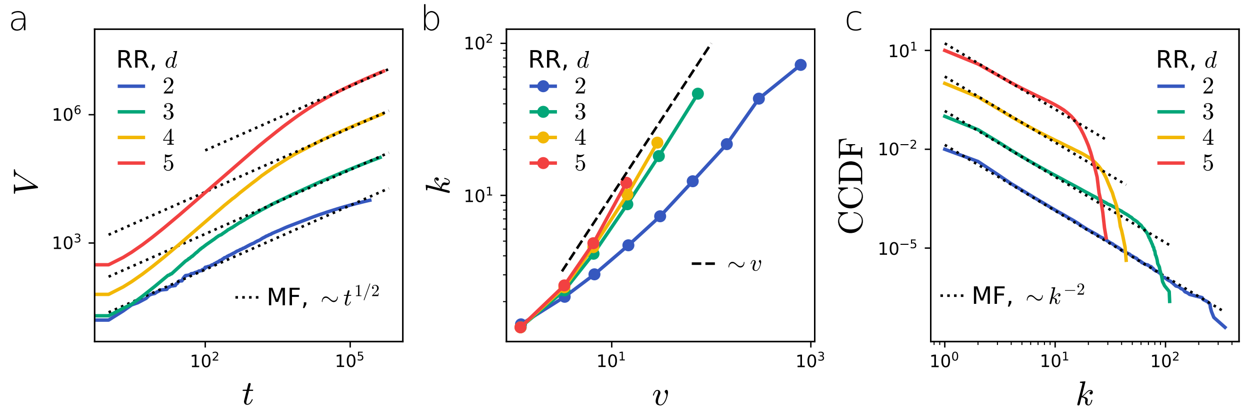

FIG. S10. **RR networks.** (a) Evolution of the total volume of the network V_t. (b) Correlations between node volume v and node degree degree k. (c) Complementary cumulative distribution of the node degree. (a-c) We compare the predictions of Eqs. (S1),(S2) and (S3) to numerical simulations, finding excellent agreement with two caveats:(i) for $d = 2$, the evolution of the total volume and the degree-volume correlations somewhat deviate from the prediction and (ii) for $d \leq 3$, there is an upper cutoff in the degree distribution. The lines indicate results for a single saturated network embedded on d-dimensional square lattice with periodic boundaries and side length L, where L is chosen such that $L^d \approx 10^6$.

S2. EMPIRICAL PHYSICAL NETWORKS

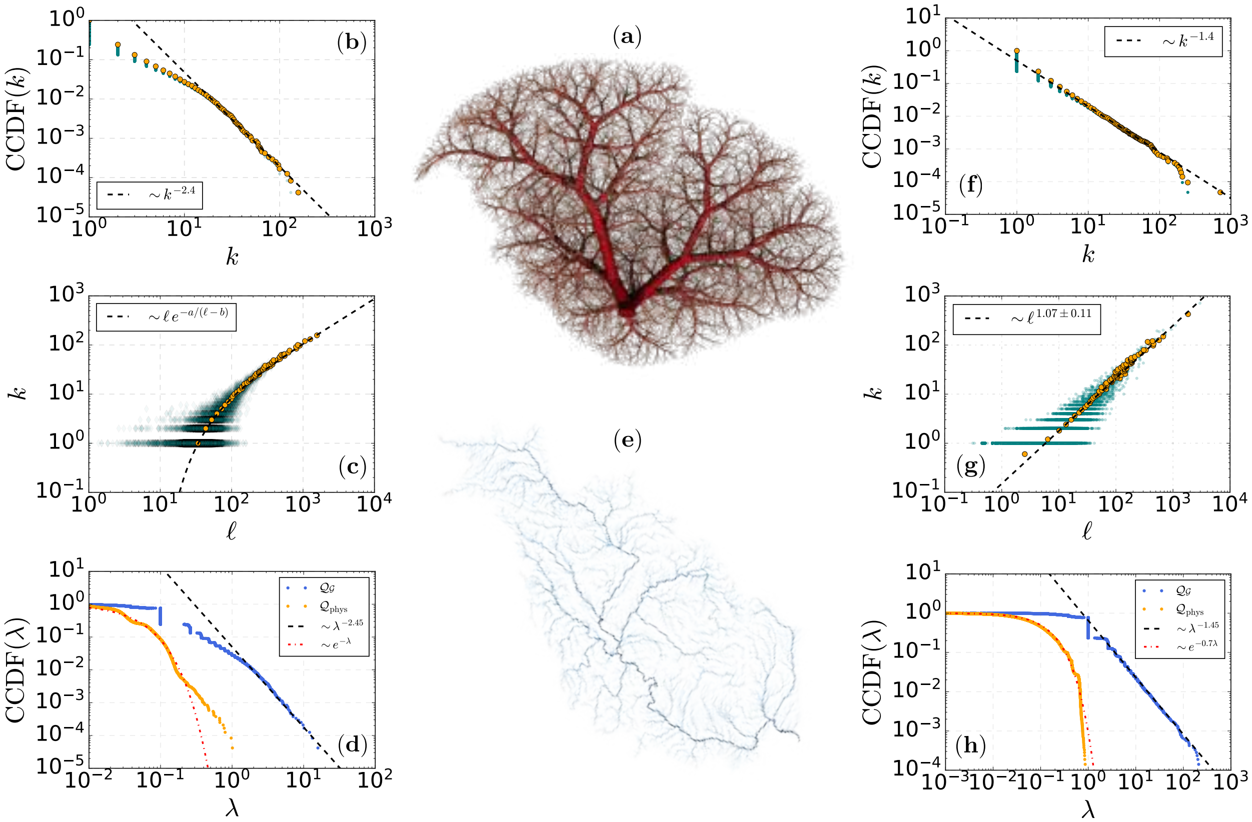

FIG. S11. **Network-of-networks analysis of real physical networks: Part I**. (**a**) Hepatic vascular network (HVN) generated from the corrosion cast (main sub-tree up to depth 5) of a human liver via geometric optimization [5]. The combinatorial graph, $\mathcal{G}$, is built following Horton's river stream ordering scheme [6] —i.e., by labeling tributaries downstream— and it consists of $N_{\text{HVN}} = 5.9 \times 10^3$ nodes and $M_{\text{HVN}} = 5.9 \times 10^3$ edges. (**b**) CCDF of the degree distribution of $\mathcal{G}$, showing a power-law tail with exponent $\gamma_{\text{HVN}} \simeq 3.4$. (**c**) Linear degree-length correlation in the HVN accompanied by an exponential cut-off below $k \sim 10$ —fitted curve with $a \simeq 50$ and $b \simeq 4$— where also the degree distribution changes trend. Given the elongated nature of physical nodes, we use lengths instead of volumes. Note that, for elongated links, positive degree-length correlation implies positive degree-volume correlations. (**d**) CCDF of the spectrum of the combinatorial Laplacian $\mathbf{Q}_{\mathcal{G}}$ (blue) and the physical Laplacian $\mathbf{Q}_{\text{phys}} = \mathbf{V}^{-1/2}\mathbf{Q}_{\mathcal{G}}\mathbf{V}^{-1/2}$ (orange) spectra, where the latter is obtained as in Eq. (7) in the main text, replacing volumes with lengths. Like in model networks, the spectrum of $\mathbf{Q}_{\mathcal{G}}$ exhibits a power-law tail with exponent close to γ_{HVN}, while for $\mathbf{Q}_{\text{phys}}$ we observe a rapidly decaying spectrum. (**e**) Danube river network (DRN); data obtained from the HydroRIVERS database [7]. Its combinatorial graph consists of $N_{\text{DRN}} = 2.1 \times 10^4$ nodes and $M_{\text{DRN}} = 2.1 \times 10^4$ edges, and physical nodes are labeled following Horton's ordering scheme. (**f**) CCDF of the DRN degree distribution, showing a power-law tail with exponent $\gamma_{\text{DRN}} \simeq 2.4$. (**g**) Degree-length correlations in the DRN; best fit returns the exponent 1.07 ± 0.11. Similar to the HVN data set, we use lengths instead of volumes. (**h**) CCDF of the DRN Laplacian spectrum of $\mathbf{Q}_{\mathcal{G}}$ (blue) decays as a power law, while the spectrum of $\mathbf{Q}_{\text{phys}}$ (orange) has an exponential tail. Results similar to (**a**)–(**d**) and in (**e**)–(**h**) are found in other synthetic vascular and river networks (not shown). To calculate the spectrum of $\mathbf{Q}_{\text{phys}}$, we measure the volume in units such that the minimum node volume is unity, i.e., $\min_i v_i = 1$.

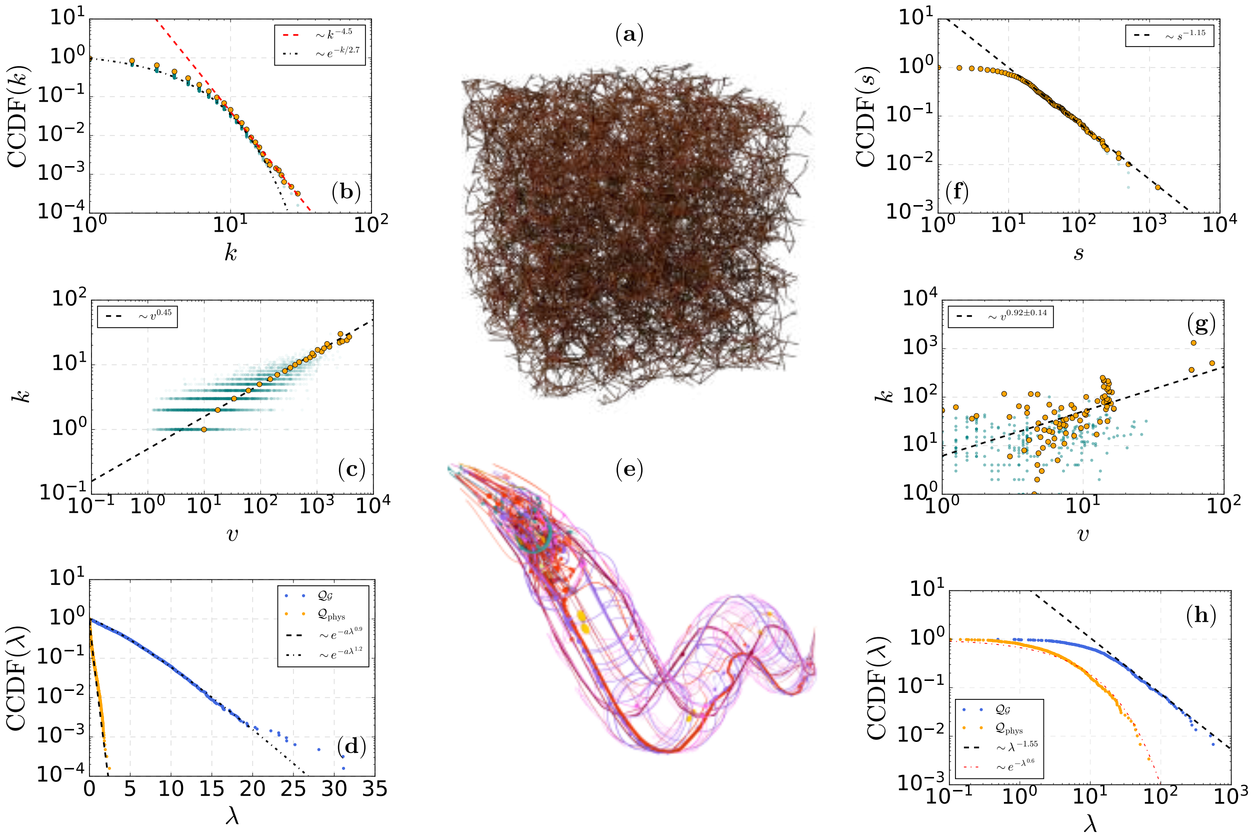

(a)
(b)
(c)
(d)
(e)
(f)
(g)
(h)
CCDF(k)
k
$\sim k^{-4.5}$
$\sim e^{-k/2.7}$
v
$\sim v^{0.45}$
CCDF(λ)
λ
$\mathcal{Q}_{\mathcal{G}}$
$\mathcal{Q}_{\rm phys}$
$\sim e^{-a\lambda^{0.9}}$
$\sim e^{-a\lambda^{1.2}}$
CCDF(s)
s
$\sim s^{-1.15}$
$\sim v^{0.92\pm0.14}$
$\sim \lambda^{-1.55}$
$\sim e^{-\lambda^{0.6}}$

FIG. S12. **Network-of-networks analysis of physical networks: Part II**. (**a**) Berea sandstone porous network (SPN) [8]. In the combinatorial graph $\mathcal{G}$, nodes represent pores –i.e. large voids in the rock– and links model narrow pathways connecting pores, called throats [9]; here $N_{\mathrm{SPN}} = 6 \times 10^3$ and $M_{\mathrm{SPN}} = 1.2 \times 10^4$. (**b**) The number of throats are homogeneously distributed as reflected by the exponential decay of the degree CCDF. (**c**) We observe positive yet sublinear degree-volume correlations. (**d**) The CCDF of both the spectrum of $\mathbf{Q}_{\mathcal{G}}$ and $\mathbf{Q}_{\mathrm{phys}}$ both exhibit a stretched exponential decay due to the homogeneous degree distribution of $\mathcal{G}$. (**e**) Neural network (NN) of an adult hermaphrodite *C. elegans*; neuron size is obtained from Ref. [10] and the combinatorial graph is from Ref. [11]. The combinatorial graph takes into account only the gap junction synapses between neurons and the network is treated as undirected multi-graph, in which case $N_{\mathrm{NN}} = 302$ and $M_{\mathrm{NN}} = 1.1 \times 10^3$. We had to drop three neurons from the analysis because their volume was missing. (**f**) CCDF of the strength degree distribution of $\mathcal{G}$, showing a power law tail with exponent $\gamma_{\mathrm{NN}} \simeq 2.2$. (**g**) Degree-volume correlations; despite that volumes are less heterogeneous then in other examples, best fit indicates a linear correlation with exponent $\alpha = 0.92 \pm 0.14$. (**h**) CCDF of the spectrum of $\mathbf{Q}_{\mathcal{G}}$ and $\mathbf{Q}_{\mathrm{phys}}$ for the *C. elegans* network. We find that spectrum of $\mathbf{Q}_{\mathcal{G}}$ has a power law tail whose exponent is in agreement with γ_{NN} and a visible exponential decay for the spectrum of spectra of $\mathbf{Q}_{\mathrm{phys}}$. To calculate the spectrum of $\mathbf{Q}_{\mathrm{phys}}$, we measure the volume in units such that the minimum node volume is unity, i.e., $\min_i v_i = 1$.

SUPPLEMENTARY REFERENCES

[1] Neal Madras and Gordon Slade. *The self-avoiding walk.* Springer Science & Business Media, 2013.

[2] Imtiaz Majid, Naeem Jan, Antonio Coniglio, and H Eugene Stanley. Kinetic growth walk: A new model for linear polymers. *Physical Review Letters*, 52(15):1257, 1984.

[3] S Hemmer and PC Hemmer. An average self-avoiding random walk on the square lattice lasts 71 steps. *The Journal of Chemical Physics*, 81(1):584–585, 1984.

[4] S Havlin and D Ben-Avraham. Corrections to scaling in self-avoiding walks. *Physical Review A*, 27(5):2759, 1983.

[5] Etienne Jessen, Marc C Steinbach, Charlotte Debbaut, and Dominik Schillinger. Rigorous mathematical optimization of synthetic hepatic vascular trees. *Journal of the Royal Society Interface*, 19(191):20220087, 2022.

[6] Amos Maritan, Andrea Rinaldo, Riccardo Rigon, Achille Giacometti, and Ignacio Rodríguez-Iturbe. Scaling laws for river networks. *Physical Review E*, 53(2):1510, 1996.

[7] Bernhard Lehner and Günther Grill. Global river hydrography and network routing: baseline data and new approaches to study the world's large river systems. *Hydrological Processes*, 27(15):2171–2186, 2013.

[8] Hu Dong. *Micro-CT imaging and pore network extraction.* PhD thesis, Department of Earth Science and Engineering, Imperial College London, 2008.

[9] Hu Dong and Martin J Blunt. Pore-network extraction from micro-computerized-tomography images. *Physical Review E*, 80(3):036307, 2009.

[10] Balázs Szigeti, Padraig Gleeson, Michael Vella, Sergey Khayrulin, Andrey Palyanov, Jim Hokanson, Michael Currie, Matteo Cantarelli, Giovanni Idili, and Stephen Larson. Openworm: an open-science approach to modeling caenorhabditis elegans. *Frontiers in computational neuroscience*, 8:137, 2014.

[11] Steven J Cook, Travis A Jarrell, Christopher A Brittin, Yi Wang, Adam E Bloniarz, Maksim A Yakovlev, Ken CQ Nguyen, Leo T-H Tang, Emily A Bayer, Janet S Duerr, et al. Whole-animal connectomes of both caenorhabditis elegans sexes. *Nature*, 571(7763):63–71, 2019.
